# Metabolomic and transcriptomic analyses highlight metabolic regulatory networks of *Salvia miltiorrhiza* in response to replant disease

**DOI:** 10.1186/s12870-024-05291-2

**Published:** 2024-06-18

**Authors:** Mei Jiang, YaXing Yan, BingQian Zhou, Jian Li, Li Cui, LanPing Guo, Wei Liu

**Affiliations:** 1 Key Laboratory for Natural Active Pharmaceutical Constituents Research in Universities of Shandong Province, School of Pharmaceutical Sciences, Qilu University of Technology (Shandong Academy of Sciences), Jinan, 250014 China; 2 Key Laboratory for Applied Technology of Sophisticated Analytical Instruments of Shandong Province, Shandong Analysis and Test Center, Qilu University of Technology (Shandong Academy of Sciences), Jinan, 250014 China; 3 National Resource Center for Chinese Materia Medica, China Academy of Chinese Medical Sciences, Beijing, 100700 China; 4 Jinan Institute of Product Quality Inspection, Jinan, 250101 China

**Keywords:** *Salvia miltiorrhiza*, Replant disease, Metabolome, Transcriptome, Plant hormone, Flavonoid

## Abstract

**Background:**

*Salvia miltiorrhiza*, a well-known traditional Chinese medicine, frequently suffers from replant diseases that adversely affect its quality and yield. To elucidate *S. miltiorrhiza*’s metabolic adaptations to replant disease, we analyzed its metabolome and transcriptome, comparing normal and replant diseased plants for the first time.

**Results:**

We identified 1,269 metabolites, 257 of which were differentially accumulated metabolites, and identified 217 differentially expressed genes. Integrated transcriptomic and metabolomic analyses revealed a significant up-regulation and co-expression of metabolites and genes associated with plant hormone signal transduction and flavonoid biosynthesis pathways in replant diseases. Within plant hormone signal transduction pathway, plants afflicted with replant disease markedly accumulated indole-3-acetic acid and abscisic acid, correlating with high expression of their biosynthesis-related genes (*SmAmidase*, *SmALDH*, *SmNCED*, and *SmAAOX3*). Simultaneously, changes in hormone concentrations activated plant hormone signal transduction pathways. Moreover, under replant disease, metabolites in the local flavonoid metabolite biosynthetic pathway were significantly accumulated, consistent with the up-regulated gene (*SmHTC1* and *SmHTC2*). The qRT-PCR analysis largely aligned with the transcriptomic results, confirming the trends in gene expression. Moreover, we identified 10 transcription factors co-expressed with differentially accumulated metabolites.

**Conclusions:**

Overall, we revealed the key genes and metabolites of *S. miltiorrhiza* under replant disease, establishing a robust foundation for future inquiries into the molecular responses to combat replant stress.

**Supplementary Information:**

The online version contains supplementary material available at 10.1186/s12870-024-05291-2.

## Background

*Salvia miltiorrhiza* Bunge, a member of the Lamiaceae family, has been celebrated for its roots, playing a pivotal role in traditional Chinese medicine for over two millennia. Renowned for its capacity to enhance blood circulation and alleviate blood stasis, growing evidence suggests that *S. miltiorrhiza* may protect against vascular diseases, notably atherosclerosis and heart disease [1]. In clinical settings, the sodium sulphate derivative of tanshinone IIA, the primary compound within *S. miltiorrhiza*, has been extensively utilised to treat patients with coronary artery disease and angina pectoris [2]. The molecular biology of *S. miltiorrhiza* has been extensively studied, with the publication of multiple versions of its genome [3, 4]. In recent years, a large number of studies have conducted comprehensive analyzes of the metabolome and transcriptome of *S. miltiorrhiza*. For example, using metabolomics and transcriptomics, it was revealed that the SmMYB36-SmERF6/SmERF115 module regulates the biosynthesis of tanshinone and phenolic acids [5], SmDXS5 plays a key regulatory role in the primary and secondary metabolism of tanshinone [6], and nitrogen starvation promotes the expression of genes involved in the MVA and MEP pathways involved in tanshinone and terpene backbone biosynthesis [7]. The combination of metabolome and transcriptome has become an effective means to identify stress-responsive and metabolism-related functional genes.

Replant disease is a pervasive agricultural challenge arising from the repeated cultivation of the same species in a particular location, leading to notable morphological and physiological alterations in affected plants [8]. This condition frequently culminates in diminished stress resistance, reduced crop yield and quality, hampered normal growth, and, in severe cases, potential widespread plant mortality [9, 10]. The cultivation of *S. miltiorrhiza* is particularly susceptible to replant disease, causing abnormal root growth and significantly impacting the yield and quality of medicinal materials. Researchers commonly attribute replant diseases to three primary mechanisms: soil nutrient imbalance, increased populations of harmful microbes, and the auto toxic effects of allelopathy [11–13]. For instance, under replant conditions, root secretions from *Rehmannia glutinosa* can promote the growth of the pathogen *Fusarium oxysporum*, exacerbating replant diseases [14]. This proliferation hinders salicylic acid signalling and fosters the onset of replant diseases [15]. Allelochemical exposure can profoundly affect plant respiration, disrupt oxidative phosphorylation, mitochondrial functionality, and ATP synthase activities, stimulate the accumulation of reactive oxygen species, and inhibit the antioxidant system of the plant. This cascade of events leads to lipid peroxidation and structural damage to the cell membrane [16–18]. Despite these findings, the molecular mechanisms by which plants sense and transducer these external conditions, resulting in the symptoms of replant disease, remain unclear.

The alteration of metabolites in plants under adverse stress primarily reflects the plant response and defence mechanisms. This adaptation results from the interplay between genes and surrounding environmental factors. Technological advancements and innovations have significantly enhanced our capacity to comprehend changes in genes and metabolites [19, 20]. Transcriptomic and metabolomic analyses have recently emerged as powerful tools for unveiling stress-response mechanisms and signal transduction pathways. For example, a combination of transcriptomic, metabolomic, and physiological analyses has illuminated the physiological and molecular mechanisms by which potassium regulates cotton root salt tolerance and the role of flavonoids in poplar resistance to poplar anthracnose [21, 22]. Metabolic alterations in response to various stresses differ significantly among different plants, and the regulatory mechanisms involved in multiple metabolic pathways are intricate [23, 24]. Research indicates that phytohormone signal transduction pathways, MAPK signal transduction pathways, and phenylpropanoid metabolism pathways are of great importance in plant responses to abiotic and biotic stresses [25–28]. Despite this progress, the primary metabolic pathways and key regulatory factors of *S. miltiorrhiza* under continuous cropping stress are still unclear.

In this study, we conducted, for the first time, a comprehensive analysis integrating a widely targeted metabolome and strand-specific transcriptome to investigate replant diseases. Our aim was to elucidate metabolite variations in response to replant disease in *S. miltiorrhiza*, identify the key metabolic pathways involved, and reveal the relationship between changes in metabolic and transcriptional levels. This study will broaden the understanding of the molecular mechanisms by which *S. miltiorrhiza* responds to replant diseases and offer insights that could inform the future breeding of resistant *S. miltiorrhiza* varieties.

## Materials and methods

### Plant materials

S. *miltiorrhiza* plants, genetically consistent and uniform in size, were cultivated in two different soils, both subjected to standardised management. The first soil, previously unused for *S. miltiorrhiza* cultivation, yielded plants labelled as normal or “N”. The second soil, employed for *S. miltiorrhiza* cultivation for one year, produced plants labelled as replant disease or “R”. For each group, 20 *S. miltiorrhiza* plants were planted. During the root expansion stage, roots from three independent plants from each soil were randomly sampled and designated as “N1”, “N2”, “N3”, “R1”, “R2”, and “R3”. These samples were immediately frozen in liquid nitrogen and stored at -80 °C. The plant sample was identified by Wei Liu. These specimens have been deposited in our lab (School of Pharmaceutical Sciences, Qilu University of Technology) with the accession numbers JM202301-JM202306).

### Metabolite extraction and UPLC-ESI-MS/MS analyses

Roots of *S. miltiorrhiza* underwent vacuum freeze-drying followed by grinding into a powder. The 50 mg sample powder was mixed with 1200 µL of a 70% methanol solution containing 2-chlorophenylalanineas an internal standard (CAS: 14091-11-3; purity: 98%; manufacturer: J&K Scientific, Beijing, China; concentration: 1PPM (mg/L)). The internal standard is added to the extract for quality control and to monitor the stability of the assay. After centrifugation, the resulting supernatant was filtered through a 0.22 μm microporous film for UPLC-ESI-MS/MS analyses (UPLC, ExionLC AD series; MS, AB Sciex 4500 Q TRAP). Chromatographic separation employed an Agilent SB-C18 column (2.1 mm × 100 mm, 1.8 μm). The mobile phase comprised two parts: A (water with 0.1% formic acid) and B (acetonitrile with 0.1% acetic acid), starting at 95% A and 5% B, transitioning to 5% A and 95% B over 9 min, maintaining this gradient for 1 min before reverting to initial conditions for 1.1 min and equilibrating for 2.9 min. The flow rate was set at 0.35 mL/min, and the sample chamber temperature was maintained at 40 °C. Each injection introduced 4 µL of the sample. ESI-QTRAP-MS operated under the following conditions: ion source temperature at 550 °C, ion spray voltage of -4500 V and 5500 V in negative and positive modes, with gas flows for curtain, I, and II at 25, 50, and 60 psi, utilising enhanced collision-induced dissociation settings.

For the qualitative analysis of metabolites, the primary and secondary MS data were used to annotate metabolites based on the selfbuilt metware database (MWDB) (Wuhan Metware Biotechnology, Wuhan, China) and the public metabolite database [29]. To ensure the accuracy of the metabolite annotations, the interference signals, including the repeated signals of K^+^, Na^+^, and NH_4_^+^ ions, the isotope signal, and the repetitive signals of fragment ions, were first excluded during the analysis. The metabolite structures were analyzed by reference to the public databases (MassBank, KNApSAcK, HMDB, MoTo DB, and METLIN). Quantitative metabolite determination occurred in multiple reaction-monitoring modes. Characteristic ions of each metabolite selectively passed through the triple quadrupole, and their signal intensities were measured using a detector. MultiQuant version (v 3.0.2) handled the integration and correction of chromatographic peaks. Finally, peak area integration represented relative metabolite amounts.

### Extraction of RNA and sequencing for transcriptomics

Total RNA extraction was performed using the Tiangen Biotech RNA isolation kit. We tested the concentration and purity of RNA using NanoDrop One spectrophotometer (NanoDrop Technologies, DE, USA) and Qubit 3.0 Fluorometer (Life Technologies, CA, USA). The integrity of the RNA was checked by agarose gel electrophoresis. Total RNA was further fragmented and ribosomal RNA degraded into purified RNA. Sequencing libraries were generated from purified RNA using NEBNext® UltraTM RNA Library Prep Kit for Illumina® (New England Biolabs, Ipswich, USA). The steps for library construction were as follows: first-strand cDNA synthesis with random primers, second-strand cDNA synthesis, and substitution of dTTP with dUTP. Following purification, A-tailing, adapter ligation, and PCR amplification, the first cDNA strand was selected for next-generation sequencing, leveraging the enzyme specificity of the Illumina platform during amplification. The constructed library was quantified using Qubit 3.0 Fluorometer (Life Technologies, CA, USA), and then detected using Agilent 2100 bioanalyzer (Agilent, CA, USA). Sequencing was conducted on a NovaSeq 6000 (Illumina, CA, USA). Library construction and sequencing were performed by Benagen company (Benagen, Wuhan, China).

The sequencing data underwent quality control using FastQC (v 0.11.9) [30]. Low-quality reads were trimmed using Fastp (v 0.21.0) [31] with default parameters. Filtered transcriptome reads were aligned to the *S. miltiorrhiza* 99 − 3 reference genome [3] through Star (v 2.7.9a) [32]. The mapped reads were assembled into transcripts with StringTie (v 2.1.4) [33] using default parameters. Gene expression levels were quantified using the RNA-Seq by Expectation Maximization (RSEM) method [34] and reported as FPKM values. Differentially expressed genes (DEGs) were identified through DESeq2 (v 1.26.0) [35] with a significance threshold of *p* < 0.05 and |log2FoldChange| ≥ 1. Subsequently, DEGs were annotated with Gene Ontology (GO) terms and Kyoto Encyclopedia of Genes and Genomes (KEGG) pathways using clusterProfiler (v. 3.14.3) [36].

### Integrated metabolomic and transcriptomic analysis

To identify statistical map of differential metabolites (DAMs) in *S. miltiorrhiza* samples under normal and replant stress conditions, we employed orthogonal partial least squares-discriminant analysis (OPLS-DA) on metabolite concentrations using MetaboAnalystR (v1.0.1). To avoid over-fitting, 200 permutation tests were conducted. Variable importance in the projection (VIP) values were extracted from the OPLS-DA results. Metabolites with VIP > 1 and |Log2FC| ≥ 1 were considered DAMs and annotated using KEGG pathways.

The correlation between metabolite concentration and gene expression in the six samples was calculated using the ‘rcorr’ function in R. A nine-quadrant diagram was drawn in R to visualise the fold difference between genes and metabolites with a Pearson correlation coefficient |r| ≥ 0.85 and *p* ≤ 0.05 for each group. The metabolites co-expressed with the genes were annotated using KEGG pathways.

### Quantitative real-time PCR (qRT-PCR) confirmation

To validate through QRT-PCR, we employed the Hifair® II 1st Strand cDNA Synthesis SuperMix kit (Yeasen Biotechnology, Shanghai, China) to transcribe total RNA into cDNA. We used the Hieff® qPCR SYBR Green Master Mix kit (Yeasen Biotechnology, Shanghai, China) with the following reaction system: 2 µl of Hieff® qPCR SYBR Green Master Mix (Low Rox Plus), 1 µl of Forward Primer (10 µM), 1 µl of Reverse Primer (10 µM), 1 µl of cDNA, and 15 µl of ddH2O. Then, QRT-PCR assays were performed on a QuanStudio5 system (Thermo Fisher Scientific, Massachusetts, USA) with the following conditions: 95 °C for 5 min; 40 cycles of 95 °C for 10 s, 60 °C for 20 s and 72 °C for 20 s; and the melting curve stage uses the instrument’s default parameters. Primers for these genes were manually designed with Primer 5.0 software. We use the *SmActin* as the endogenous reference as documented previously [37]. To calculate the relative expression of genes, we calculated the efficiencies varying between experimental and *SmActin*’s primers. If efficiencies varying by less than 10% the Livak method can be used [38]; if the experimentally established efficiencies vary by more than 10%, a correction should be made using the a Pfaffl mathematical model [39]. Correlation analysis between QRT-PCR results and RNA-Seq expression data was conducted using Python’s Pearson correlation method (v 2.7.12).

### Identification of transcription factors (TFs)

The PlantTFDB database [40] was employed for predicting TFs. Subsequently, correlation assessments between TFs and metabolites, TFs, and structural genes, as well as structural genes and metabolites, were performed using the ‘rcorr’ function in R. Only those three combinations satisfying |r| ≥ 0.85 and *p* ≤ 0.05 were screened out. Cytoscape (v 3.10.1) [41] was used to visualise the correlation networks linking TFs with structural genes.

## Results

### Metabolome profiling of the normal and replant-diseased *S. miltiorrhiza*

**Fig. 1:**
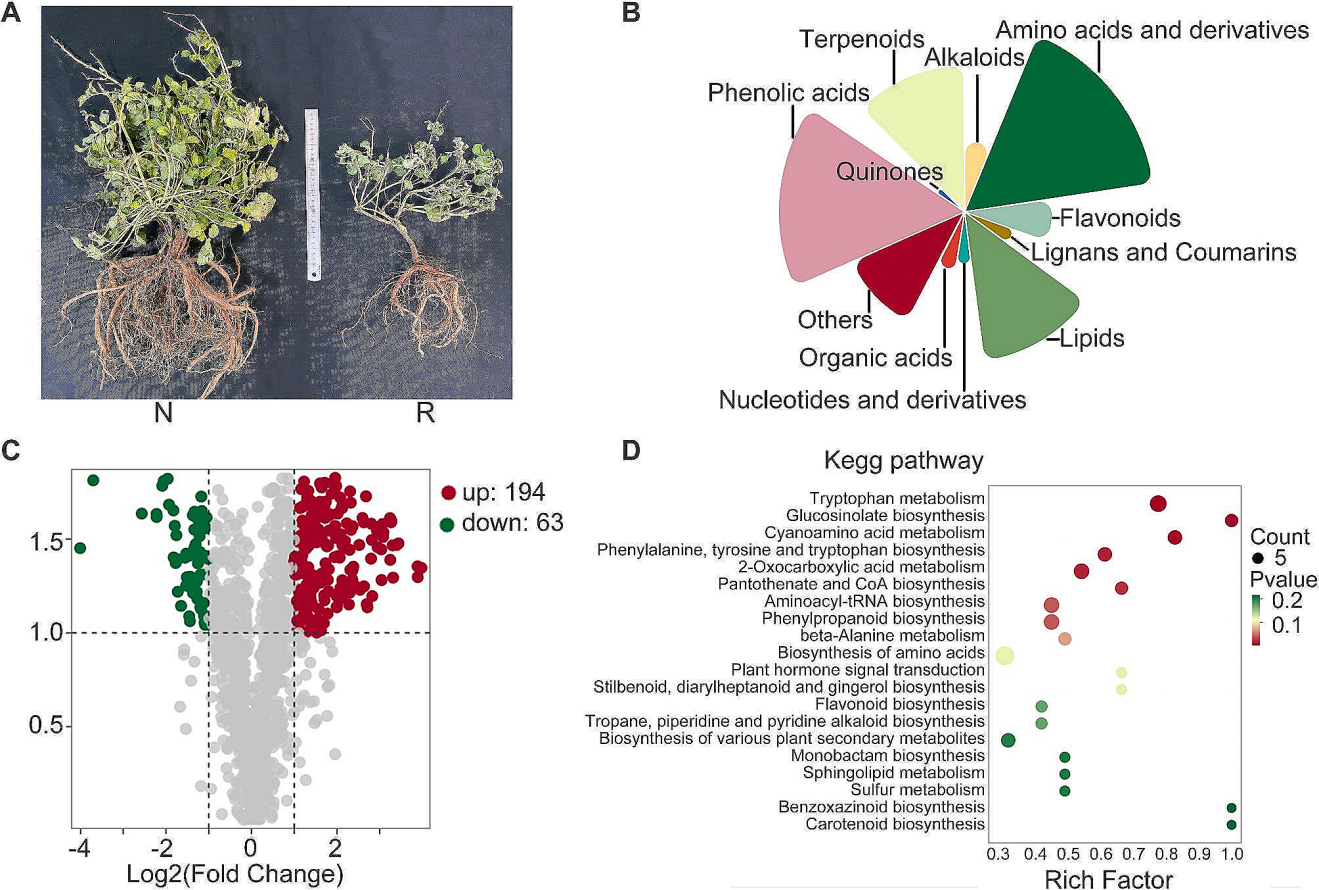
Metabolome profiling of the normal and replant diseased *S. miltiorrhiza*. (**A**) Normal and replant diseased *S. miltiorrhiza* plants. (**B**) The distribution map of metabolite types. Different colors represent different types of metabolites. The size of the graph represents the number of metabolites. (**C**) The volcano plot of differentially accumulated metabolites (DAMs). The X-axis represents the log2 (fold change) value, and the Y-axis represents the VIP value. Each point represents a metabolite. Green represents the metabolite’s log2 (fold change) ≤ -1 and VIP ≥ 1. Red represents the metabolite’s log2 (fold change) ≥ 1 and VIP ≥ 1. (**D**) The KEGG pathway analysis of DAMs. Based on the *p* value, the top 20 KEGG pathways were displayed. Each point represents a type of KEGG pathway. The point size reflect the metabolite count within that pathway, while coloration reflect the *p* value

*S. miltiorrhiza* plants were collected during root expansion. In comparison with normal controls, the roots afflicted with replant disease exhibited noticeable growth stunting (Fig. 1A). A comprehensive targeted metabolomic analysis was conducted on both normal and replant-diseased roots using UPLC-MS/MS. A total of 1,269 metabolites spanning 11 categories were successfully detected and quantitatively measured, as outlined in Table S1. The results revealed consistent metabolite distribution profiles between the two root types, with amino acids and their derivatives constituting the most abundant category (17%), followed by phenolic acids (16%), lipids (13%), terpenoids (13%), flavonoids (8%), and alkaloids (6%) (Fig. 1B). The OPLS-DA results demonstrated significant differences in metabolite concentrations between the normal and replant-diseased roots (Fig. S1).

A total of 257 differentially accumulated metabolites (DAMs) were identified using a threshold of |Log2FC| ≥1 and VIP ≥ 1. Among these, 63 were highly expressed in normal roots, while 194 were highly expressed in replant-diseased roots (Fig. 1C). KEGG pathway analysis annotated these DAMs into various pathways, including tryptophan metabolism, glucosinolate biosynthesis, cyanoamino acid metabolism, phenylalanine, tyrosine, tryptophan biosynthesis, 2-oxocarboxylic acid metabolism, pantothenate and CoA biosynthesis, aminoacyl-tRNA biosynthesis, phenylpropanoid biosynthesis, beta-alanine metabolism, biosynthesis of amino acids, plant hormone signal transduction, stilbenoid, diarylheptanoid and gingerol biosynthesis, and flavonoid biosynthesis. (Fig. 1D, Table S2).

### Transcriptome profiling of the normal and replant-diseased *S. miltiorrhiza*

**Fig. 2:**
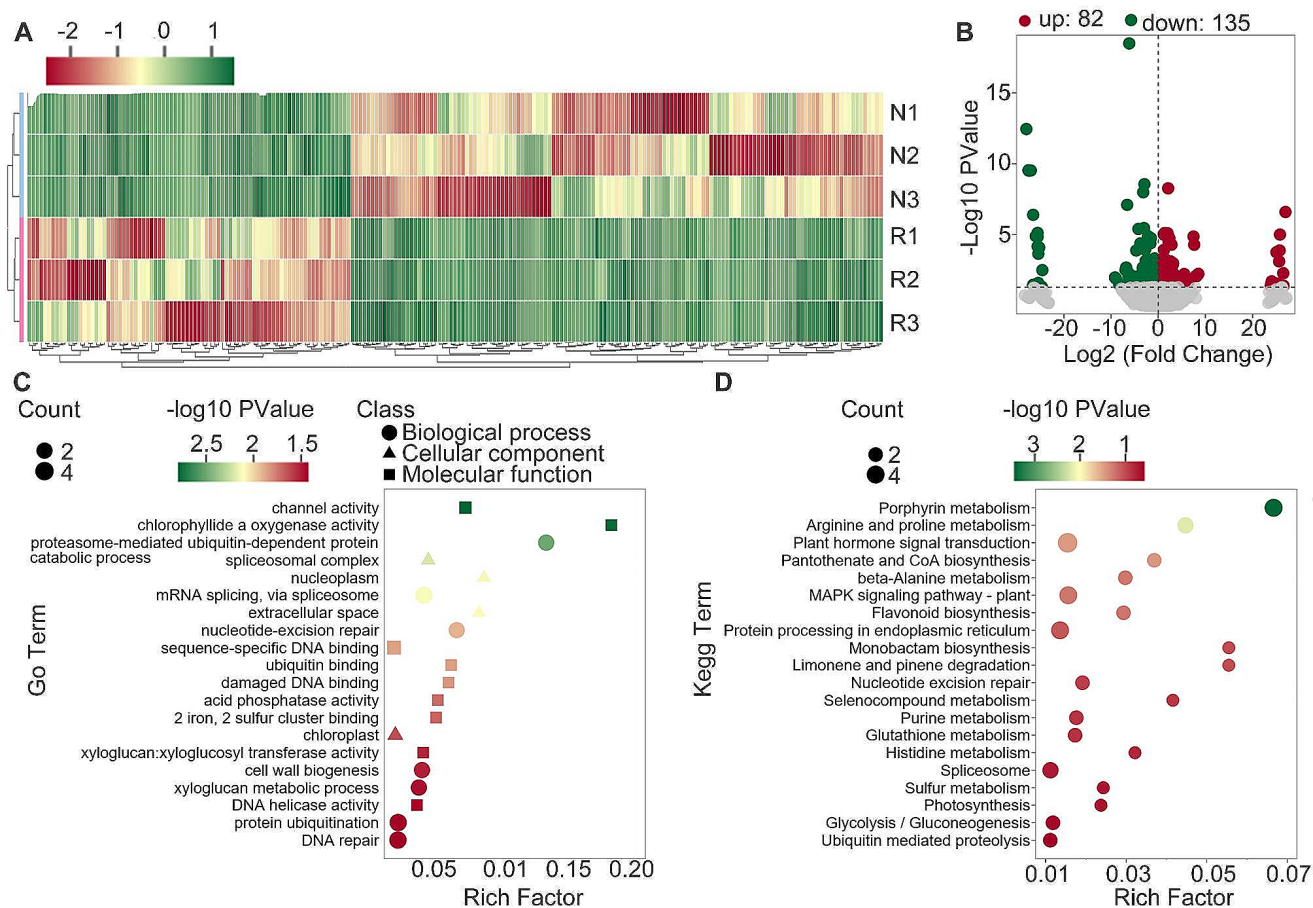
Transcriptome profiling of the normal and replant diseased *S. miltiorrhiza*. (**A**) Clustering heat map of differentially expressed genes (DEGs). Each row represents a sample, and each column represents a gene. The color corresponds to the Z-score transformed from the FPKM values of gene. (**B**) The volcano plot of DEGs. Points with log2 (fold change) ≤ 1 and *p* < 0.05 represent significantly down-regulated genes, shown in green. Points withlog2 (fold change) ≥ 1 and *p* < 0.05 represent significantly up-regulated genes, shown in red. (**C**) Analysis of Go terms for DEGs. Based on the *p* value, the top 20 GO terms were displayed. Each row represents a Go term. The circle, triangle and rectangle represent the biological process, cellular component, and molecular function, respectively. The point size represents the gene number, and the color represents the *p* value. (**D**) Analysis of KEGG for DEGs. Based on the *p* value, the top 20 KEGG pathways were displayed

To identify genes responsive to replant disease, we conducted strand-specific transcriptome sequencing on both normal and replant-diseased *S. miltiorrhiza* roots. The raw data underwent filtration, resulting in approximately 40 million reads per sample (Table S3). Subsequently, we assembled and quantified 22,224 genes based on these reads (Table S4). A comparison of the two root categories revealed expressional variance, identifying 217 DEGs (Fig. 2A). Among these, 135 exhibited high expression in normal roots, while 82 were highly expressed in diseased roots (Fig. 2B). Further analysis of GO terms and KEGG pathways was performed on these DEGs. The results indicated that the most annotated GO term was “sequence-specific DNA binding”, involving five genes. This was followed by “Chloroplast”, “ATP hydrolysis activity”, and “defence response to fungi”, each associated with four genes (Fig. 2C). KEGG pathway analysis categorised these differentially expressed genes into Porphyrin metabolism, arginine and proline metabolism, plant hormone signal transduction, Pantothenate and CoA biosynthesis, beta-alanine metabolism, MAPK signalling pathway, and flavonoid biosynthesis (Fig. 2D, Table S5). Our analysis also highlighted 19 KEGG pathways shared between DAMs and DEGs (Fig. S2), suggesting a potential collaborative role in the plant’s response to replant disease.

### Integrated analysis of DAMs and DEGs

**Fig. 3:**
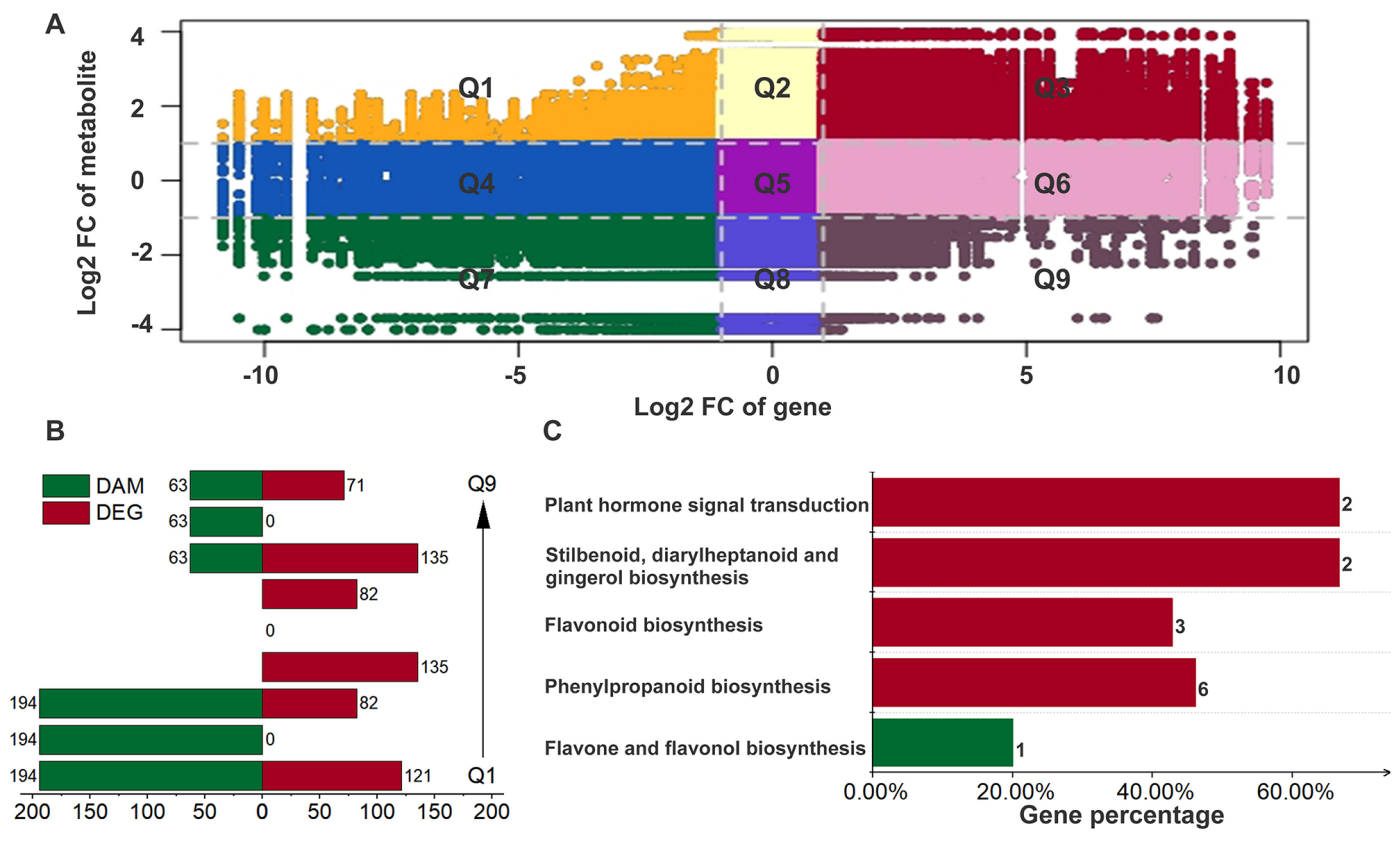
Integrated analysis of DAMs and DEGs in *S. miltiorrhiza*. (**A**) The nine-quadrant map of metabolites and genes. Each point represents a pair of correlated metabolites and genes with |r| ≥ 0.85 and *p* value ≤ 0.05. The X-axis represents the log2 (fold change) of the gene, and the Y-axis represents the log2 (fold change) of the metabolite. (**B**) Number of DAMs and DEGs in each quadrant. Each row represents a quadrant, corresponding to Q1 to Q9 in (**A**) from bottom to top. Green represents DAMs and red represents DEGs. (**C**) The KEGG analysis of metabolites. Red represents metabolites in quadrant 3. Green represents metabolites in quadrant 7. The X-axis represents the proportion of metabolites in Q3 or Q7 to the total metabolites identified on the pathways

To investigate the interplay between metabolites and gene expression, we conducted a correlation analysis of their respective patterns and illustrated the outcomes using a nine-quadrant diagram (Fig. 3A). Most metabolites and genes were situated in quadrants 2, 4, 6, and 8, indicating that while many genes and metabolites were relevant, they did not exhibit a response to replant disease. Notably, metabolites and genes in quadrants 3 and 7 exhibited a positive correlation.

Quadrant 3 encompassed 225 metabolites (including 194 DAMs) and 1,377 genes (including 82 DEGs) whose expression levels significantly increased in response to replant disease (Fig. 3B). In contrast, quadrant 7 included 71 metabolites (63 DAMs) and 1,846 genes (135 DEGs) with significantly down-regulated expression levels in replant disease. Subsequently, KEGG enrichment analysis was conducted for both DAMs and DEGs specifically located in quadrants 3 and 7 (Fig. 3C). In quadrant 3, we identified up-regulation in plant hormone signal transduction (ko04075) and three flavonoid metabolite biosynthesis pathways: stilbenoid, diarylheptanoid, and gingerol biosynthesis (ko00945); flavonoid biosynthesis (ko00941); and phenylpropanoid biosynthesis (ko00940). For quadrant 7, we identified pathways related to flavone and flavanol biosynthesis (ko00944). These findings suggest a significant up-regulation and co-expression of metabolites and genes associated with phytohormone signalling and flavonoid biosynthetic pathways in replant diseases.

### DAMs and DEGs involved in the pathway of plant hormone’s biosynthesis and signal transduction

**Fig. 4:**
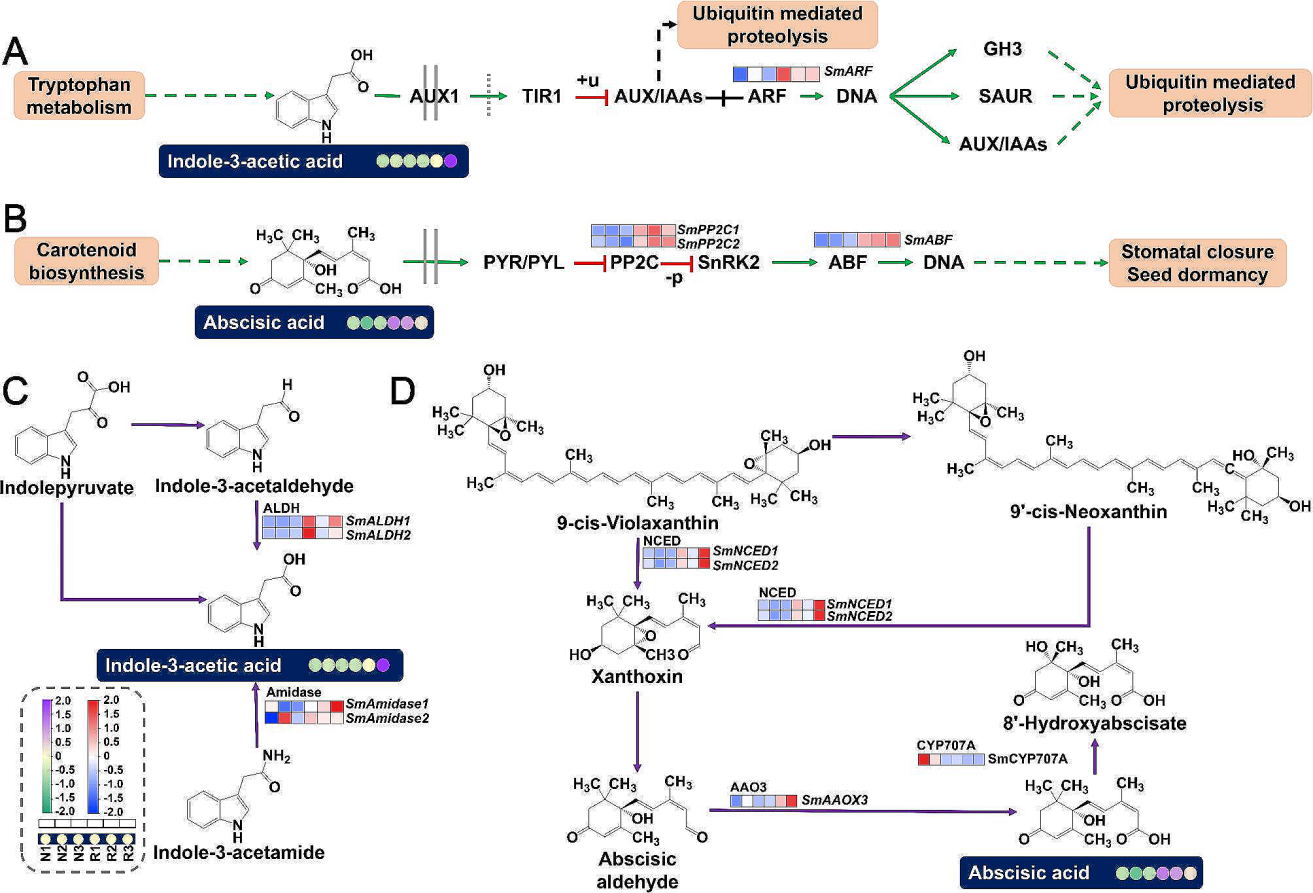
Expression of DAMs and DEGs in the pathway of plant hormone’s signal transduction and biosynthesis. The indole acetic acid (**A**) and abscisic acid (**B**) signal transduction pathways. The indole acetic acid (**C**) and abscisic acid (**D**) biosynthesis pathways. The DAMs and DEGs were tagged in the pathway. Metabolite expression level was represented by colored circles. Gene expression level was represented by colored squares. The color corresponds to the Z-score transformed from the values of metabolite or gene

The metabolome analysis revealed a significant increase in hormone concentrations, particularly indole-3-acetic acid and abscisic acid, under replant stress. Transcriptome KEGG analysis indicated substantial enrichment of DEGs within the plant hormone signal transduction pathway (ko04075). The concentration of indole-3-acetic acid in replant-diseased *S. miltiorrhiza* significantly increased with a fold change of 9.9, compared to the control group. Figure 4A illustrates this pathway, the expression of the auxin response factor (*ARF*) gene (*SmARF*) was up-regulated, while other genes in this pathway showed no notable changes. Further exploration of the indole-3-acetic acid biosynthesis pathway (ko00380) unveiled up-regulation of specific genes (*SmAmidase1*) encoding amidase and aldehyde dehydrogenase (*ALDH*) genes (*SmALDH1* and *SmALDH2*) under replant stress (Fig. 4C). Both amidase and ALDH catalyse the synthesis of Indole-3-acetate acid from Indole-3-acetamide and Indole-3-acetaldehyde, respectively.

The concentration of abscisic acid in replant-diseased *S. miltiorrhiza* significantly increased with a fold change of 2.2 compared to the control group. Figure 4B illustrates the abscisic acid-mediated signal transduction pathway, indicating a significant up-regulation of two type 2 C protein phosphatases genes (*SmPP2C1 and SmPP2C2*) in replant-diseased *S. miltiorrhiza*. The expression levels of these genes showed a significant positive correlation with abscisic acid concentration (*r* = 0.94, *p* = 0.0060 and *r* = 0.81, *p* = 0.0483). Furthermore, the up-regulation of the ABA-responsive element binding factors gene (*SmABF*) correlated significantly with the abscisic acid concentration (*r* = 0.85, *p* = 0.0316). We also observed up-regulation of some genes in the abscisic acid biosynthetic pathway under replant disease, including two nine-cis-epoxycarotenoid dioxygenase genes (*SmNCED*1 and *SmNCED*2) and one abscisic aldehyde oxidase 3 gene (*SmAAOX3*) (Fig. 4D). *NCED* catalyses the conversion of 9-*cis*-violaxanthin to xanthoxin, which then generates abscisic aldehyde. *AAOX3* catalyses the conversion of abscisic aldehydes to abscisate acids. Generally, under replant disease conditions, *S. miltiorrhiza* roots activate plant hormone biosynthetic pathways, leading to the accumulation of plant hormones. Simultaneously, changes in hormone concentrations activate plant hormone signal transduction pathways, thereby regulating downstream biological processes.

### DAMs and DEGs involved in the flavonoid biosynthetic pathway

**Fig. 5:**
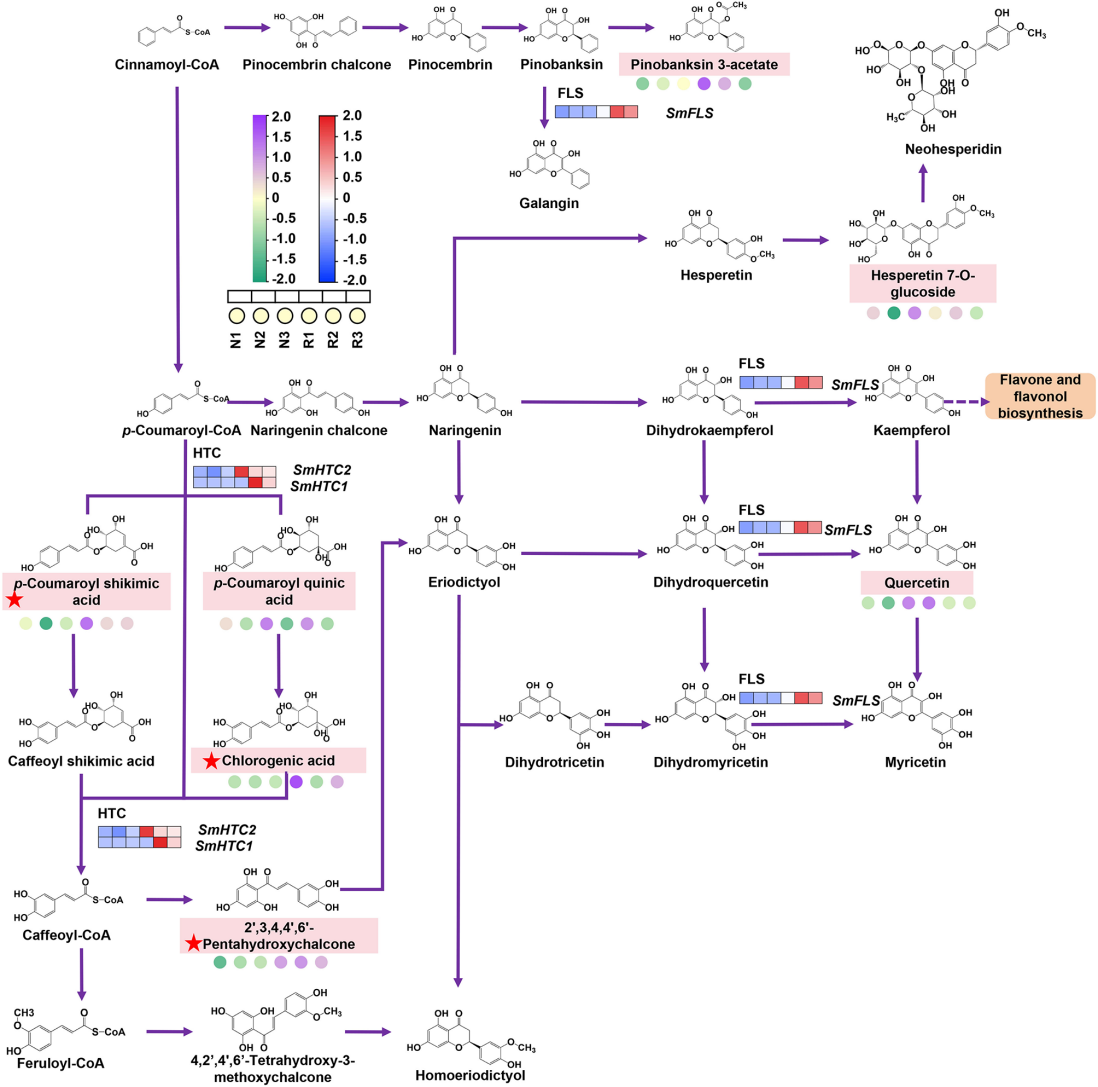
Expression of DAMs and DEGs in the flavonoid biosynthetic pathway. The metabolites identified in the flavonoid biosynthetic pathway were tagged and those that were DAMs were tagged with a red star. Metabolite expression level was represented by colored circles. Gene expression level was represented by colored squares. The color corresponds to the Z-score transformed from the values of metabolite or gene

The results of metabolomic and transcriptomic analyses indicate enrichment of the flavonoid biosynthesis pathway (ko00941) among DAMs and DEGs. In *S*. *miltiorrhiza* roots, metabolomics revealed seven compounds in the flavonoid biosynthetic pathway: chlorogenic acid (cpd_ID: C00852); 2’,3,4,4’,6’-pentahydroxychalcone (cpd_ID: C15525); *p*-coumaroyl shikimic acid (cpd_ID: C02947); hesperetin 7-O-glucoside (cpd_ID: C16422); quercetin (cpd_ID: C00389); pinobanksin 3-acetate (cpd_ID: C16418); and *p*-coumaroyl quinic acid (cpd_ID: C12208) (Fig. 5). Notably, the concentrations of chlorogenic acid, 2’,3,4,4’,6’-pentahydroxychalcone, and *p*-coumaroyl shikimic acid significantly increased under continuous cropping conditions, with fold changes of 3.3, 3.0, and 3.9, respectively. Transcriptomics identified two DEGs in the flavonoid biosynthetic pathway: the shikimate O-hydroxycinnamoyltransferase genes (*SmHTC1* and *SmHTC2*) and the flavanol synthase gene (*SmFLS*). Their expression levels were significantly up-regulated in replant disease conditions. *HCT* catalyses consecutive reactions in the flavonoid biosynthetic pathway, converting *p*-coumaroyl-CoA to *p*-coumaroyl shikimic acid and *p*-coumaroyl quinic acid. These compounds further transform into Caffeoyl shikimic acid, chlorogenic acid, and eventually 2’,3,4,4’,6’-pentahydroxychalcone through a series of enzymatic steps. The expression of *HCT* genes aligned with the accumulation of downstream metabolites, and both were significantly up-regulated under continuous cropping conditions. Flavonoids play a crucial role in plant responses to various environmental stressors. Our findings demonstrate that replant diseases activate the local flavonoid biosynthetic pathway (*p*-coumaroyl-CoA → *p*-coumaroyl quinic acid (*p*-coumaroyl shikimic acid) → chlorogenic acid (caffeoyl shikimic acid) → caffeoyl-CoA → 2’,3,4,4’,6’-pentahydroxychalcone), with significant up-regulation of two key enzyme genes in this pathway. This up-regulation promotes an increase in the concentration of metabolites within this pathway.

### Verification of gene expression by qRT-PCR

**Fig. 6:**
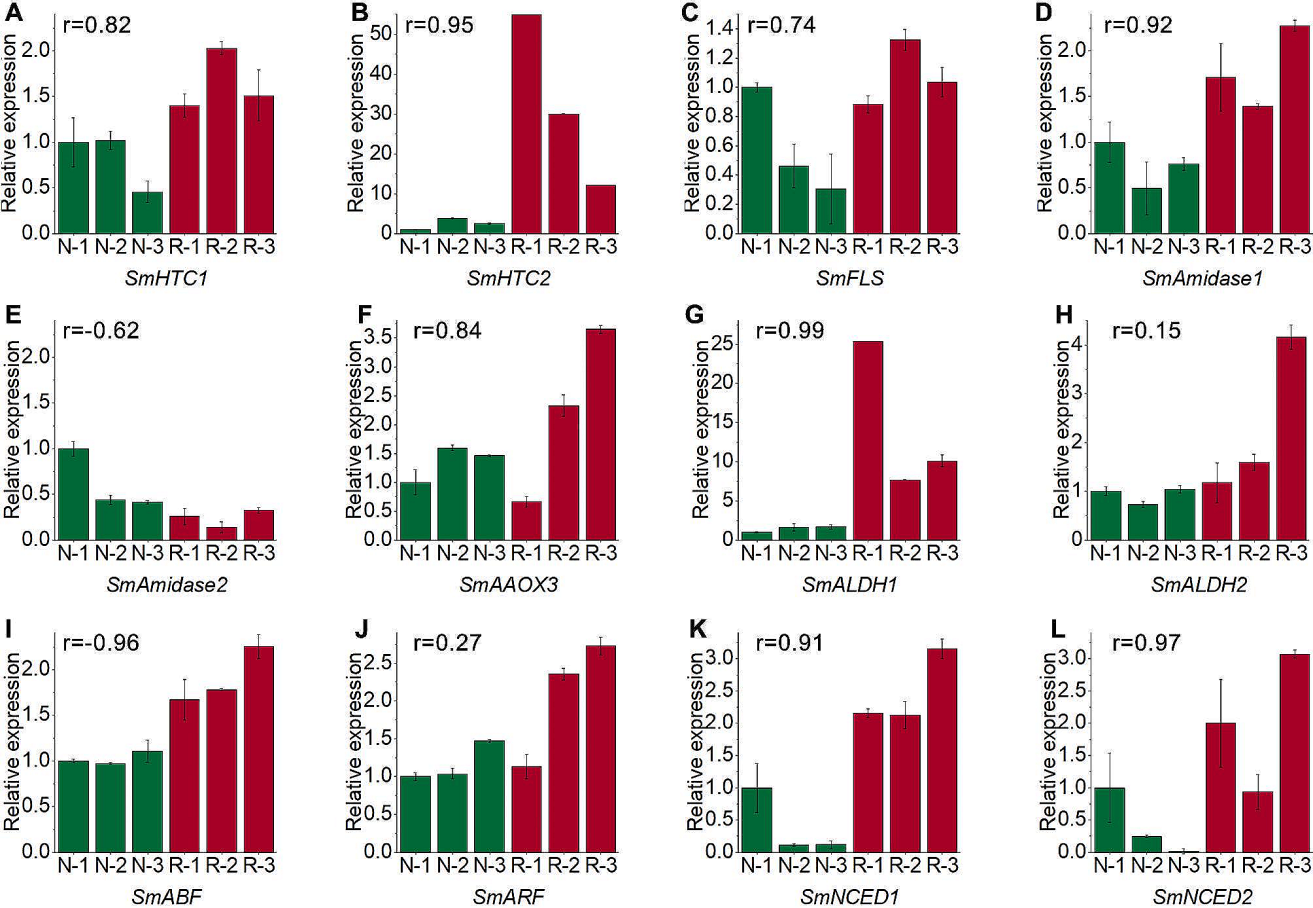
Verification of gene expression by qRT-PCR in normal and replant diseased *S. miltiorrhiza*. The Y-axis represents the relative expression of the gene. The efficiency of qRT-PCR reaction for each pair of primers were showed in Table S7. The green column represents normal *S. miltiorrhiza*, and the red column represents replant disease *S. miltiorrhiza*. The r value on each graph represents the correlation of gene expression calculated by qRT-PCR analysis and RNA-Seq methods

To validate gene expression levels based on RNA-Seq data, we conducted qRT-PCR experiments with three replicates for each sample. The primer sequences are provided in Supplementary Table S6. Twelve DEGs involved in plant hormone biosynthesis, signal transduction, and flavonoid biosynthesis pathways were chosen for validation. The results revealed up-regulation of genes associated with the indole-3-acetic acid and abscisic acid biosynthetic pathways, including *SmALDH1*, *SmALDH2*, *SmAmidase1*, *SmNCED1*, *SmNCED2*, and *SmAAO3*. Additionally, genes participating in plant hormone signal transduction pathways, such as *SmARF* and *SmABF*, along with genes in the flavonoid biosynthetic pathway, such as *SmHTC1, SmHTC2* and *SmFLS*, were up-regulated under continuous cropping conditions. Generally, except for *SmALDH2* and *SmARF*, the gene expression profiles obtained through qRT-PCR exhibited a high degree of similarity to those derived from the RNA-Seq analysis (Fig. 6).

### The co-expression network analysis of TFs and DAMs

**Fig. 7:**
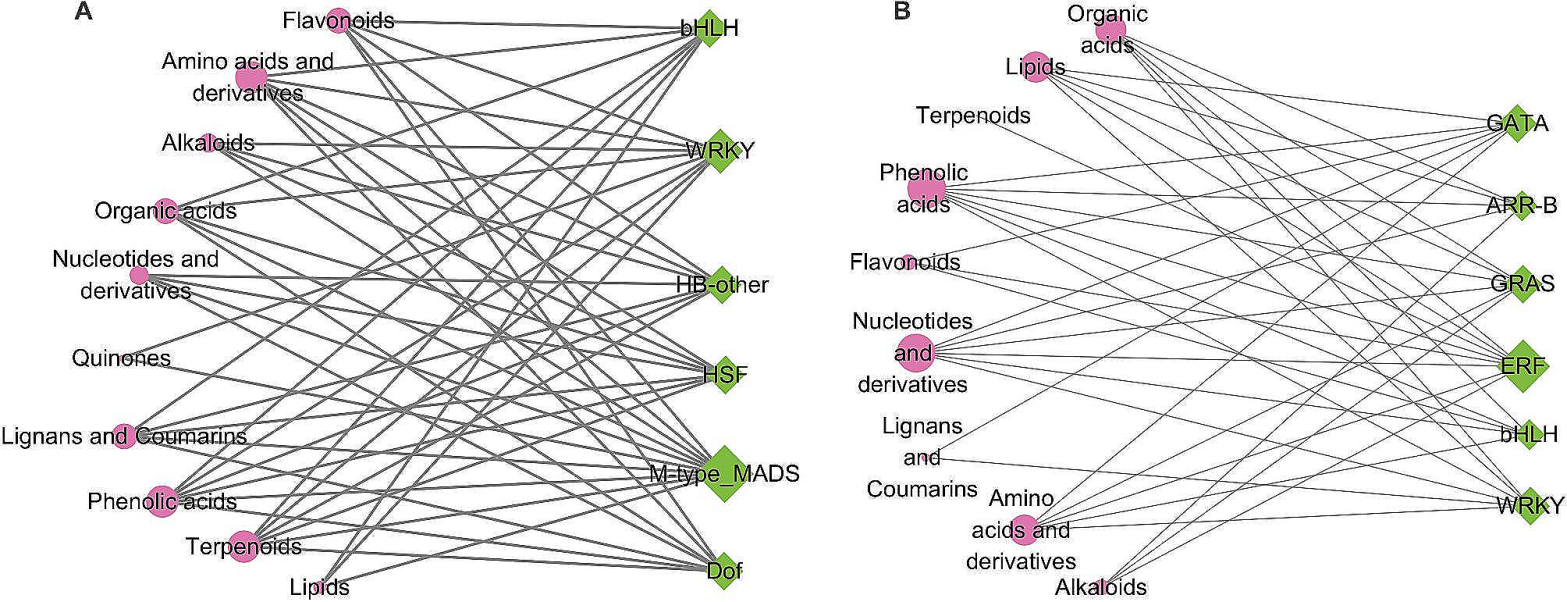
Correlation network of TFs and DAMs based on Pearson correlation. Correlation networks of TFs and DAMs up-regulated (**A**) and down-regulated (**B**) under replant disease condition. Pink circles represent DAMs and green diamonds represent TFs. Pairs of TFs and DAMs with significant positive correlation (|*r*| ≥ 0.85 and *p* ≤ 0.05) were connected by a line. The size of the circles and diamonds represents the edge count

The transcription factors (TFs) play a pivotal role in the management of stress arising from adversity. In the *S. miltiorrhiza* genome, we identified 817 TFs, encompassing 20 distinct types (Fig. S3). To investigate the relationship between TFs and DAMs under normal and replant diseased conditions, we performed co-expression network analysis of different families of DAMs and putative TFs (|*r*| ≥ 0.85 and *p* ≤ 0.05) (Fig. 7). The results showed that significantly up-regulated DMAs and significantly down-regulated ERF under replant disease had the most correlated DAMs. The other identified TFs were bHLH, Dof, WRKY, HB, GRAS, HSF, ARR-B, and GATA. These TFs were highly correlated with amino acids and derivatives, lipids, phenolic acids, quinones, terpenoids, alkaloids, flavonoids, organic acids, lignans and coumarins, and nucleotides and derivatives. For example, changes in the levels of flavonoids were highly correlated with changes in the levels of transcription factors bHLH, WRKY, HB, MADS, and Dof. In summary, these results suggest that the transcriptional regulatory network mediated by TFs including MADS, ERF, bHLH, Dof, WRKY, HB, GRAS, HSF, ARR-B, and GATA has a potential function in regulating the replant response of *S. miltiorrhiza*.

## Discussion

The diseases associated with replantation hinder the normal growth and metabolic functions of *S. miltiorrhiza*, significantly impacting its yield and quality. Metabolites form the biochemical foundation of plant phenotypic variation, exhibiting a wide diversity in stress responses [42]. The reconstruction of the metabolome under stress is a crucial reflection of adaptive and defensive strategies of a plant [43, 44]. In this study, we conducted a comprehensive, widely targeted metabolomic analysis of the roots of *S. miltiorrhiza* grown in both non-continuous and continuous cropping soils, identifying a total of 1,269 metabolites. The OPLS-DA plot illustrated a distinct separation between the sample groups, indicating significant differences in secondary metabolite composition attributed to varying soil conditions. In field experiments, using plant pools (10 or 20 plants in one pool) to represent an experimental sample can more accurately reflect the effects of replanting diseases on plant growth and secondary metabolism. However, in this experiment, we need to conduct correlation analysis between metabolome and transcriptome, so we chosen to use one plant to represent the experimental sample to reflect the one-to-one correspondence between the metabolic content and gene expression in a single plant.

Previous studies on replanting diseases in various species have primarily focused on specific metabolite types or rhizosphere soil metabolites. For example, changes in the concentrations of seven metabolites were compared in roses grown in different soils, and root exudates in response to ginsenoside stress were detected in *Panax notoginseng* [45, 46]. However, a comprehensive exploration of widely targeted metabolomic alterations within plants in response to replantation diseases remains unexplored. Consequently, this study provides valuable insights into the potential of metabolome remodelling across different species when confronted with replanting diseases. Soil degradation caused by persistent soil-borne pathogens, including bacteria, fungi, and nematodes, often initiates plant diseases. Concurrently, the accumulation of auto toxic chemicals around the roots significantly contributes to these diseases [47–49]. Adverse conditions can induce changes in the concentrations and activities of plant hormones, subsequently affecting physiological processes [50]. Our findings reveal a notable increase in the levels of indole acetic acid and abscisic acid in *S. miltiorrhiza* roots affected by replant disease compared to normal roots. Simultaneously, the expression of genes involved in the biosynthetic pathways of these two hormones significantly increased, including *ALDH* and Amidase genes for indole acetic acid synthesis, and additional *NCED* and *AAO3* genes for abscisic acid biosynthesis. Conversely, the expression of genes within the abscisic acid catabolism pathway decreased in *S. miltiorrhiza* with replant disease. This suggests that, under stress, *S. miltiorrhiza* roots respond by modulating gene expression to promote the accumulation of indole acetic acid and abscisic acid. In this experiment, the expression levels of identified genes differed among individuals of *S. miltiorrhiza* plants. We believe that this is normal and that differences in gene expression levels may be caused mainly by genomic differences between individuals. Although we showed the gene expression profiles of each individual in the figure, we used the average of three biological replicates in each treatment group for differential expression analysis.

Indole acetic acid (IAA) plays a crucial role as an auxin, influencing plant growth and development [51]. Key TFs involved in auxin signal transduction include ARFs and AUX/IAA inhibitors. The AUX/IAA family proteins inhibit the expression of auxin-responsive genes, while ARFs can either suppress or promote the expression of downstream genes. In the context of replant disease, expression of *SmARF* gene was up-regulated, highlighting a complex mechanism for auxin regulation of root growth and development under replant stress [52]. Abscisic acid (ABA) is an essential hormone that governs plant responses to stress and influences various aspects of plant development, such as seed sprouting, root architecture, ageing, and seed maturation [53]. For example, ABA can promote auxin biosynthesis, thereby inhibiting primary root elongation in rice [54]. We observed a significantly higher concentration of abscisic acid in *S. miltiorrhiza* affected by replant disease, accompanied by a substantial elevation in the expression of genes encoding key enzymes in its downstream signal transduction pathway, namely *SmPP2C* and *SmABF*. The PP2C-PYR/PYL/RCAR complex and AREB/ABF-SnRK2 are highly conserved abscisic acid signal transduction pathways that positively regulate abscisic acid/stress signalling [55]. Under unfavourable conditions, plants may produce more abscisic acid, facilitating bonding between PYR/PYL and PP2C, leading to the dissociation of the SnRK2-PP2C-SnRK1 complex, activation of SnRK1, inhibition of target of rapamycin activity, and suppression of growth [56]. We hypothesise that in *S. miltiorrhiza* under replant disease stress, the increased expression of abscisic acid synthesis genes and the substantial accumulation of abscisic acid may inhibit root growth and development. Further research is necessary to fully elucidate these complex signalling mechanisms and their impact on plant health and resilience.

Flavonoids, a class of polyphenolic compounds, constitute key secondary metabolites in plants [57, 58]. They serve various functions, including antioxidant activity, ultraviolet protection, and defence against both biotic and abiotic stresses [58]. In our current investigation, we identified 98 flavonoid compounds in *S. miltiorrhiza*. Existing research suggests that environmental stress can stimulate the synthesis and accumulation of specific flavonoids in plants. For example, under mild drought stress, *Senna obtusifolia* exhibits significant accumulation of naringenin and emodin. Similarly, in response to salt stress, sorghum demonstrates increased concentrations of flavonoids along with enhanced expression of their biosynthetic genes [23, 59]. We observed similar phenomena in replant-diseased *S. miltiorrhiza*. Not only did the concentration of flavonoids change significantly, but we also noted significant alterations in the upstream compounds and genes within the flavonoid biosynthetic pathway. Noteworthy compounds include 2’,3,4,4’,6’-pentahydroxychalcone, its precursor chlorogenic acid, and *p*-Coumaroyl shikimic acid. These changes are significant in the context of replant diseases, which involve intricate interactions among plants, auto toxic substances, and microorganisms. Both chlorogenic acid and 2’,3,4,4’,6’-pentahydroxychalcone are known for their antimicrobial activity [60, 61]. Chlorogenic acid exhibits antifungal properties against plant pathogenic fungi such as *Fusarium nucleatum*, *Colletotrichum capsici*, *Alternaria dianthi*, *Botrytis cinerea*, and *Cercospora sojina*, completely preventing spore germination or inhibiting fungal growth [62–64]. In *S. miltiorrhiza* affected by replant disease, we observed a substantial accumulation of chlorogenic acid and pentahydroxyflavones. This accumulation may play an inhibitory role against pathogenic microorganisms, and further experimental validation is warranted.

## Conclusions

To the best of our knowledge, this study represents the first comprehensive analysis of the metabolome and transcriptome of *S. miltiorrhiza*. We elucidated the intricate relationship between changes at the metabolite and transcript levels during the plant’s response to replant disease. Notably, our findings indicate the activation of two key pathways—plant hormone signal transduction and flavonoid metabolite biosynthesis—under replant disease stress. Metabolites and genes associated with these biosynthetic and signal transduction pathways exhibited significant up-regulation. This foundational research deepens our insights into the roles of hormones and flavonoids in replant diseases and provides valuable information for the selection of new, resistant varieties of *S. miltiorrhiza*.

### Electronic supplementary material

Below is the link to the electronic supplementary material.

Supplementary Material 1

Supplementary Material 2

## Data Availability

The raw data of RNA-seq has been submitted to the NCBI database with the accession numbers were SRR27032941 and SRR27032942.

## Declarations

### Permission for material collection

We are licensed to collect the plant of *Salvia miltiorrhiza* from Jinan City, Shandong Province, China. The collection complies with relevant institutional, national, and international guidelines and legislation.

## References

[1] Li ZM, Xu SW, Liu PQ (2018). Salvia miltiorrhizaBurge (Danshen): a golden herbal medicine in cardiovascular therapeutics. Acta Pharmacol Sin.

[2] Gao S, Liu Z, Li H, Little PJ, Liu P, Xu S (2012). Cardiovascular actions and therapeutic potential of tanshinone IIA. Atherosclerosis.

[3] Xu H, Song J, Luo H, Zhang Y, Li Q, Zhu Y, Xu J, Li Y, Song C, Wang B (2016). Analysis of the genome sequence of the Medicinal Plant Salvia miltiorrhiza. Mol Plant.

[4] Ma Y, Cui G, Chen T, Ma X, Wang R, Jin B, Yang J, Kang L, Tang J, Lai C (2021). Expansion within the CYP71D subfamily drives the heterocyclization of tanshinones synthesis in Salvia miltiorrhiza. Nat Commun.

[5] Qi L, Xin F, Ying Z, Ruizhi C, Juane D, Pengda MJHR. The SmMYB36-SmERF6/SmERF115 module regulates the biosynthesis of tanshinones and phenolic acids in salvia miltiorrhiza hairy roots. 2023;10. 10.1093/hr/uhac238 PMC9832864 36643739

[6] Da-Chuan Z, Ling-Long L, Zhi-Rong W, Wen-Juan X, Jun-Ling L, Shu-Ting T, Jia-Hui W, Yan L, Chi Z, Chen L et al. SmDXS5, acting as a molecular valve, plays a key regulatory role in the primary and secondary metabolism of tanshinones in Salvia miltiorrhiza. 2022;13. 10.3389/fpls.2022.1043761 PMC9685628 36438137

[7] Li-Lan L, Yu-Xiu Z, Yan-Fang YJPO. Integrative transcriptomic and metabolomic analyses unveil tanshinone biosynthesis in Salvia miltiorrhiza root under N starvation stress. 2022;17. 10.1371/journal.pone.0273495 PMC9409544 36006940

[8] Mahnkopp F, Simon M, Lehndorff E, Pätzold S, Wrede A, Winkelmann T (2018). Induction and diagnosis of apple replant disease (ARD): a matter of heterogeneous soil properties?. Sci Hort.

[9] Grunewaldt-Stöcker G, Mahnkopp F, Popp C, Maiss E, Winkelmann T (2019). Diagnosis of apple replant disease (ARD): microscopic evidence of early symptoms in fine roots of different apple rootstock genotypes. Sci Hort.

[10] Lucas M, Balbín-Suárez A, Smalla K, Vetterlein D (2018). Root growth, function and rhizosphere microbiome analyses show local rather than systemic effects in apple plant response to replant disease soil. PLoS ONE.

[11] Yang C, Xie Z, Qian S, Zhang J, Yu Z, Li M, Gu L, Qin S, Zhang Z. Functional analysis of Rehmannia Glutinosa key LRR-RLKs during interaction of root exudates with Fusarium oxysporum reveals the roles of immune proteins in formation of replant disease. Front Plant Sci. 2022;13. 10.3389/fpls.2022.1044070 PMC9660255 36388607

[12] Ajeethan N, Ali S, Fuller KD, Abbey L, Yurgel SN (2023). Apple Root Microbiome as Indicator of Plant Adaptation to Apple Replant Diseased soils. Microorganisms.

[13] Nematode–Microbe Complexes in Soils Replanted with Apple. Microorganisms. 2022;10(1):157. 10.3390/microorganisms10010157 PMC8780120 35056606

[14] Li Z, Yang Y, Wu L, Shu Y, Zhao Y, Huang W, Zhang Z, Lin W (2013). Isolation of highly pathogenic pathogens and identification of formae speciales of Rehmannia glutinosa L. Zhongguo Shengtai Nongye Xuebao/Chinese J Eco-Agriculture.

[15] Li ZF, He CL, Wang Y, Li MJ, Dai YJ, Wang T, Lin W (2016). Enhancement of trichothecene mycotoxins of Fusarium oxysporum by ferulic acid aggravates oxidative damage in Rehmannia Glutinosa Libosch. Sci Rep.

[16] Abrahim D, Francischini AC, Pergo EM, Kelmer-Bracht AM, Ishii-Iwamoto EL (2003). Effects of α-pinene on the mitochondrial respiration of maize seedlings. Plant Physiol Biochem.

[17] Hejl AM, Koster KL (2004). Juglone disrupts Root plasma membrane H+-ATPase activity and impairs Water Uptake, Root respiration, and growth in soybean (Glycine max) and corn (Zea mays). J Chem Ecol.

[18] Cheng F, Cheng Z (2015). Research Progress on the use of Plant Allelopathy in Agriculture and the physiological and ecological mechanisms of Allelopathy. Front Plant Sci.

[19] Urano K, Kurihara Y, Seki M, Shinozaki K (2010). Omics’ analyses of regulatory networks in plant abiotic stress responses. Curr Opin Plant Biol.

[20] Todd EV, Black MA, Gemmell NJ (2016). The power and promise of RNA-seq in ecology and evolution. Mol Ecol.

[21] Ju F, Pang J, Sun L, Gu J, Wang Z, Wu X, Ali S, Wang Y, Zhao W, Wang S (2023). Integrative transcriptomic, metabolomic and physiological analyses revealed the physiological and molecular mechanisms by which potassium regulates the salt tolerance of cotton (Gossypium hirsutum L.) roots. Ind Crops Prod.

[22] Zhang L, Bao H, Meng F, Ren Y, Tian C (2023). Transcriptome and metabolome reveal the role of flavonoids in poplar resistance to poplar anthracnose. Ind Crops Prod.

[23] Huang X, Chu G, Wang J, Luo H, Yang Z, Sun L, Rong W, Wang M (2023). Integrated metabolomic and transcriptomic analysis of specialized metabolites and isoflavonoid biosynthesis in Sophora alopecuroides L. under different degrees of drought stress. Ind Crops Prod.

[24] Li M, Yang Y, Feng F, Zhang B, Chen S, Yang C, Gu L, Wang F, Zhang J, Chen A (2017). Differential proteomic analysis of replanted Rehmannia glutinosa roots by iTRAQ reveals molecular mechanisms for formation of replant disease. BMC Plant Biol.

[25] Dong NQ, Lin HX (2021). Contribution of phenylpropanoid metabolism to plant development and plant-environment interactions. J Integr Plant Biol.

[26] Chen X, Ding Y, Yang Y, Song C, Wang B, Yang S, Guo Y, Gong Z (2021). Protein kinases in plant responses to drought, salt, and cold stress. J Integr Plant Biol.

[27] Danquah A, de Zelicourt A, Colcombet J, Hirt H (2014). The role of ABA and MAPK signaling pathways in plant abiotic stress responses. Biotechnol Adv.

[28] Janda T, Szalai G, Pál M (2020). Salicylic acid signalling in plants. Int J Mol Sci.

[29] Chen W, Gong L, Guo Z, Wang W, Zhang H, Liu X, Yu S, Xiong L, Luo J (2013). A Novel Integrated Method for large-scale detection, identification, and quantification of widely targeted metabolites: application in the study of Rice Metabolomics. Mol Plant.

[30] Davis EM, Sun Y, Liu Y, Kolekar P, Shao Y, Szlachta K, Mulder HL, Ren D, Rice SV, Wang Z (2021). SequencErr: measuring and suppressing sequencer errors in next-generation sequencing data. Genome Biol.

[31] Chen S, Zhou Y, Chen Y, Gu J (2018). Fastp: an ultra-fast all-in-one FASTQ preprocessor. Bioinformatics.

[32] Dobin A, Davis CA, Schlesinger F, Drenkow J, Zaleski C, Jha S, Batut P, Chaisson M, Gingeras TR (2013). STAR: ultrafast universal RNA-seq aligner. Bioinformatics.

[33] Pertea M, Pertea GM, Antonescu CM, Chang TC, Mendell JT, Salzberg SL (2015). StringTie enables improved reconstruction of a transcriptome from RNA-seq reads. Nat Biotechnol.

[34] Li B, Dewey CN (2011). RSEM: accurate transcript quantification from RNA-Seq data with or without a reference genome. BMC Bioinformatics.

[35] Love MI, Huber W, Anders S (2014). Moderated estimation of Fold change and dispersion for RNA-seq data with DESeq2. Genome Biol.

[36] Yu G, Wang LG, Han Y, He QY (2012). clusterProfiler: an R package for comparing biological themes among gene clusters. Omics.

[37] Yang Y, Hou S, Cui G, Chen S, Wei J, Huang L (2010). Characterization of reference genes for quantitative real-time PCR analysis in various tissues of Salvia miltiorrhiza. Mol Biol Rep.

[38] Livak KJ, Schmittgen TD (2001). Analysis of relative gene expression data using real-time quantitative PCR and the 2(-Delta Delta C(T)) method. Methods (San Diego Calif).

[39] Pfaffl MW (2001). A new mathematical model for relative quantification in real-time RT-PCR. Nucleic Acids Res.

[40] Jin J, Tian F, Yang DC, Meng YQ, Kong L, Luo J, Gao G (2017). PlantTFDB 4.0: toward a central hub for transcription factors and regulatory interactions in plants. Nucleic Acids Res.

[41] Shannon P, Markiel A, Ozier O, Baliga NS, Wang JT, Ramage D, Amin N, Schwikowski B, Ideker T (2003). Cytoscape: a software environment for integrated models of biomolecular interaction networks. Genome Res.

[42] Hamany Djande CY, Pretorius C, Tugizimana F, Piater LA, Dubery IA (2020). Metabolomics: A Tool for Cultivar phenotyping and investigation of grain crops. Agronomy.

[43] Peng B, Li H, Peng XX (2015). Functional metabolomics: from biomarker discovery to metabolome reprogramming. Protein Cell.

[44] Obata T, Fernie AR (2012). The use of metabolomics to dissect plant responses to abiotic stresses. Cell Mol Life Sci.

[45] Yim B, Baumann A, Grunewaldt-Stöcker G, Liu B, Beerhues L, Zühlke S, Sapp M, Nesme J, Sørensen SJ, Smalla K et al. Rhizosphere microbial communities associated to rose replant disease: links to plant growth and root metabolites. Hortic Res. 2020;7. 10.1038/s41438-020-00365-2 PMC7459328 32922816

[46] Deng L, Luo L, Li Y, Wang L, Zhang J, Zi B, Ye C, Liu Y, Huang H, Mei X (2023). Autotoxic ginsenoside stress induces changes in Root exudates to Recruit the Beneficial Burkholderia Strain B36 as revealed by transcriptomic and metabolomic approaches. J Agric Food Chem.

[47] Ilyas N, Yang Y, Liu W, Li X, Pu W, Singh RP, Li Y (2021). First report of bacterial rot caused by Pantoea Endophytica on tobacco in Liuyang, China. Plant Dis.

[48] Song W, Guo M, Zhou Y, Liang C (2019). First report of cauliflower root rot caused by Pythium coloratum in China. Plant Dis.

[49] Huang L-F, Song L-X, Xia X-J, Mao W-H, Shi K, Zhou Y-H, Yu J-Q (2013). Plant-soil feedbacks and soil sickness: from mechanisms to application in agriculture. J Chem Ecol.

[50] Verma V, Ravindran P, Kumar PP (2016). Plant hormone-mediated regulation of stress responses. BMC Plant Biol.

[51] Waadt R, Seller CA, Hsu P-K, Takahashi Y, Munemasa S, Schroeder JI (2022). Plant hormone regulation of abiotic stress responses. Nat Rev Mol Cell Biol.

[52] Kato H, Mutte SK, Suzuki H, Crespo I, Das S, Radoeva T, Fontana M, Yoshitake Y, Hainiwa E, van den Berg W (2020). Design principles of a minimal auxin response system. Nat Plants.

[53] Chen K, Li G-J, Bressan RA, Song C-P, Zhu J-K, Zhao Y (2020). Abscisic acid dynamics, signaling, and functions in plants. J Integr Plant Biol.

[54] Qin H, Wang J, Zhou J, Qiao J, Li Y, Quan R, Huang R (2023). Abscisic acid promotes auxin biosynthesis to inhibit primary root elongation in rice. Plant Physiol.

[55] Fujita Y, Yoshida T, Yamaguchi-Shinozaki K (2013). Pivotal role of the AREB/ABF-SnRK2 pathway in ABRE-mediated transcription in response to osmotic stress in plants. Physiol Plant.

[56] Belda-Palazón B, Adamo M, Valerio C, Ferreira LJ, Confraria A, Reis-Barata D, Rodrigues A, Meyer C, Rodriguez PL (2020). Baena-González E: a dual function of SnRK2 kinases in the regulation of SnRK1 and plant growth. Nat Plants.

[57] Dixon RA, Achnine L, Kota P, Liu C-J, Reddy MSS, Wang L (2002). The phenylpropanoid pathway and plant defence—a genomics perspective. Mol Plant Pathol.

[58] Wang L, Chen M, Lam P-Y, Dini-Andreote F, Dai L, Wei Z (2022). Multifaceted roles of flavonoids mediating plant-microbe interactions. Microbiome.

[59] Ma S, Lv L, Meng C, Zhang C, Li Y (2020). Integrative analysis of the Metabolome and Transcriptome of Sorghum bicolor reveals dynamic changes in Flavonoids Accumulation under saline-alkali stress. J Agric Food Chem.

[60] Saqallah FG, Hamed WM, Talib WH, Dianita R, Wahab HA (2022). Antimicrobial activity and molecular docking screening of bioactive components of Antirrhinum majus (snapdragon) aerial parts. Heliyon.

[61] Martínez G, Regente M, Jacobi S, Del Rio M, Pinedo M, de la Canal L (2017). Chlorogenic acid is a fungicide active against phytopathogenic fungi. Pestic Biochem Physiol.

[62] Sung WS, Lee DG (2010). Antifungal action of chlorogenic acid against pathogenic fungi, mediated by membrane disruption. Pure Appl Chem.

[63] Niggeweg R, Michael AJ, Martin C (2004). Engineering plants with increased levels of the antioxidant chlorogenic acid. Nat Biotechnol.

[64] Shetty R, Fretté X, Jensen B, Shetty NP, Jensen JD, Jørgensen HJL, Newman M-A, Christensen LP (2011). Silicon-induced changes in antifungal phenolic acids, flavonoids, and key phenylpropanoid pathway genes during the interaction between miniature roses and the biotrophic pathogen Podosphaera pannosa. Plant Physiol.

