# Supplementary material for: Metabolomic and transcriptomic analyses highlight metabolic regulatory networks of Salvia miltiorrhiza in response to replant disease
Source: BMC Plant Biol. 2024 Jun 18;24:575. doi: 10.1186/s12870-024-05291-2 (PMC11184839; doi:10.1186/s12870-024-05291-2)
Supplement: Supplementary file 1 — Supplementary Material 1 [file 12870_2024_5291_MOESM1_ESM.docx]

# Supplementary Figures

**Title**

**Metabolomic and transcriptomic analyses highlight metabolic regulatory networks of *Salvia miltiorrhiza* in response to replant disease**

Mei Jiang^1,2^, YaXing Yan^2^, BingQian Zhou^1,2^, Jian Li^4^, Li Cui^1,2^, LanPing Guo^3^，Wei Liu^1,2,*^

**Affiliations**

^1^ Key Laboratory for Natural Active Pharmaceutical Constituents Research in Universities of Shandong Province, School of Pharmaceutical Sciences, Qilu University of Technology (Shandong Academy of Sciences), Jinan, 250014, China

^2^ Key Laboratory for Applied Technology of Sophisticated Analytical Instruments of Shandong Province, Shandong Analysis and Test Center, Qilu University of Technology (Shandong Academy of Sciences), Jinan, 250014, China

^3^ National Resource Center for Chinese Materia Medica, China Academy of Chinese Medical Sciences, Beijing, 100700, China

^4^ Jinan Institute of Product Quality Inspection, Jinan, 250101, China

*** Corresponding author**

Wei Liu, liuwei0074@163.com

**
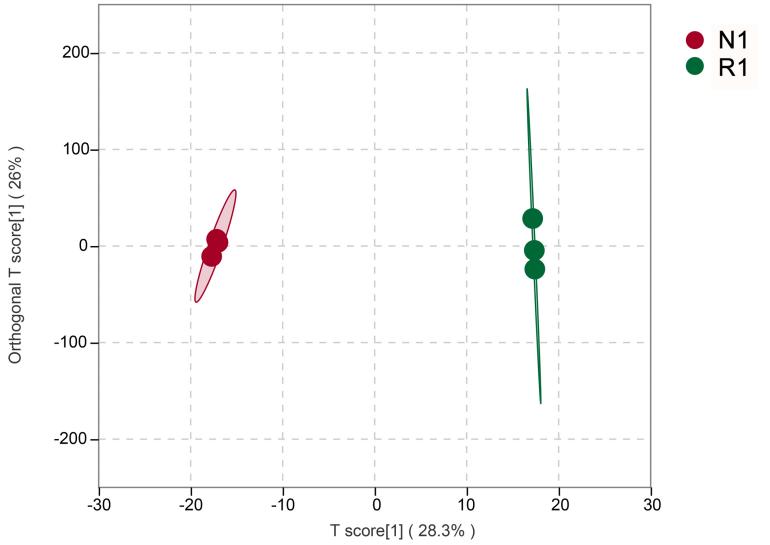
**

**Fig. S1 The OPLS-DA score plot.**

The X-axis represents the predicted principal component, and the abscissa direction shows the gap between groups. The Y-axis represents the orthogonal principal component, and the ordinate direction shows the gap within the group. The percentage represents the explanation rate of the component for the data set. Each point in the figure represents a sample, and samples in the same group are represented by the same color.


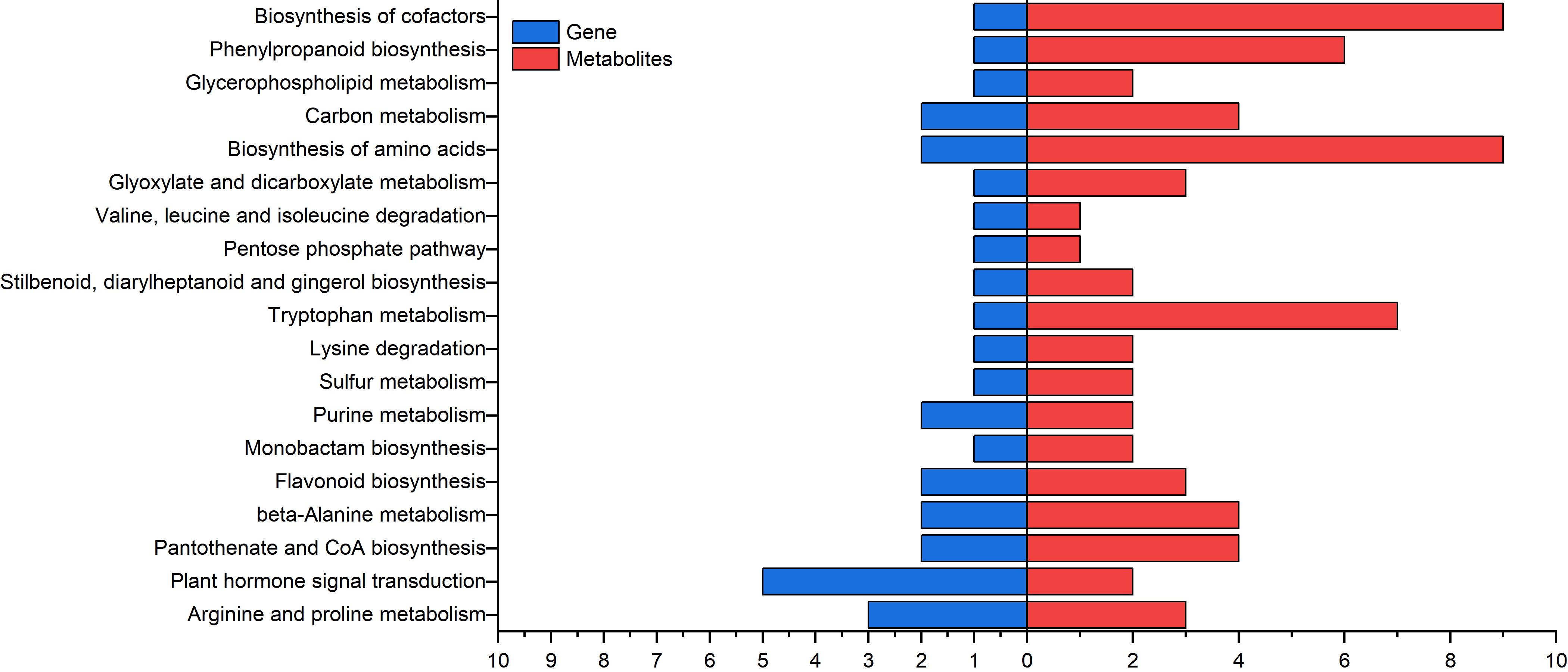


**Fig. S2 The intersection of KEGG pathways annotated by both DAMs and DEGs.**

Each row represents a KEGG pathway, with blue indicating DAMs and red indicating DEGs. The X-axis represents the number of DAMs or DEGs annotated within each pathway.


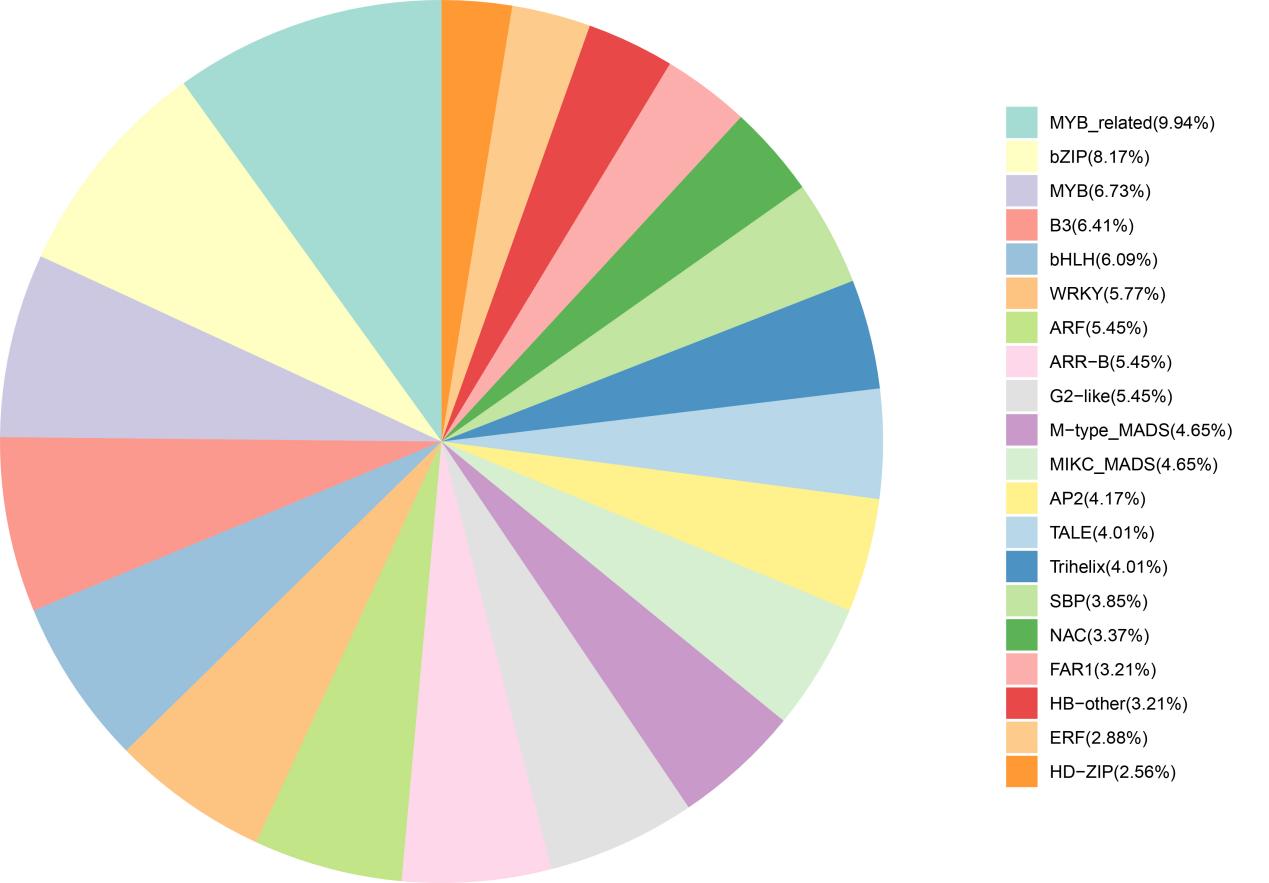


**Fig. S3 The proportion map of transcription factor family .**

This map employs diverse colors to denote different transcription factor families. The numbers on the legend represent the proportion of each type of transcription factor.
